# Sinus Fungus Ball in the Japanese Population: Clinical and Imaging Characteristics of 104 Cases

**DOI:** 10.1155/2013/731640

**Published:** 2013-11-12

**Authors:** Kazuhiro Nomura, Daiya Asaka, Tsuguhisa Nakayama, Tetsushi Okushi, Yoshinori Matsuwaki, Tsuyoshi Yoshimura, Mamoru Yoshikawa, Nobuyoshi Otori, Toshimitsu Kobayashi, Hiroshi Moriyama

**Affiliations:** ^1^Department of Otorhinolaryngology, Jikei University School of Medicine, 3-25-8 Nishishinbashi, Minato-ku, Tokyo 105-8461, Japan; ^2^Department of Otolaryngology-Head and Neck Surgery, Tohoku University Graduate School of Medicine, 1-1 Seiryo-cho, Aoba-ku, Sendai, Miyagi 980-8574, Japan

## Abstract

Sinus fungus ball is defined as noninvasive chronic fungal rhinosinusitis occurring in immunocompetent patients with regional characteristics. The clinical and imaging characteristics of paranasal sinus fungus ball were retrospectively investigated in 104 Japanese patients. All patients underwent endoscopic sinus surgery. Preoperative computed tomography (CT), magnetic resonance (MR) imaging, age, sex, chief complaint, causative fungus, and clinical outcome were analyzed. Patients were aged from 25 to 79 years (mean 58.8 years). Female predominance was noted (58.7%). Most common symptoms were nasal discharge and facial pain. CT showed high density area in 82.0% of the cases (82/100), whereas T2-weighted MR imaging showed low intensity area in 100% of the cases (32/32). Histological examination showed that most causative agents were *Aspergillus* species (94.2% (98/104)). Culture test was positive for 16.7% (11/66). Recurrence was found in 3.2% (3/94). Older age and female predominance were consistent with previous reports. MR imaging is recommended to confirm the diagnosis.

## 1. Introduction

Fungal rhinosinusitis is encountered in about 10% of patients requiring surgery for diseases of the nose and sinuses, and fungal or mixed fungal and bacterial infections are responsible for 13.5% to 28.5% of all cases of maxillary sinusitis [1, 2]. Sinus fungus ball is a form of fungal sinusitis defined as noninvasive chronic fungal sinusitis without inspissated allergic mucin and occurs in immunocompetent hosts. The clinical condition now defined as “fungus ball” was previously called “mycetoma,” “aspergillosis,” or “aspergilloma,” but better understanding of its pathophysiology has led to an update of terminology by recommending the use of the term “fungus ball” [1, 2]. Surgical treatment with the endoscope usually results in good outcome.

Several case series have been reported [2–6], but none in the Japanese population. Fungal infection is reported to have regional characteristics. Only 30 of 109 patients lived in urban areas with a population greater than 50,000 [5]. A study of the composition of the ambient air showed different fungus species present in France and the USA [7]. This study analyzed cases of sinus fungus ball in the Japanese population.

## 2. Materials and Methods

We retrospectively reviewed the clinical records of patients diagnosed with sinus fungus ball who underwent surgery at the Department of Otorhinolaryngology, Jikei University Hospital, Tokyo, Japan, between April 2005 and November 2010. The diagnosis was based on histological examination of the surgically removed material. Patients diagnosed with invasive fungal sinusitis or allergic fungal sinusitis (AFS) were excluded. We analyzed age, sex, chief complaint, location of the fungus ball, presence of high density area on computed tomography (CT), presence of low intensity area on T2-weighted magnetic resonance (MR) imaging, causative fungus, and surgical outcome. Good outcome is defined as opening of the operated sinus. Patients were followed up for 6 months postoperatively. The institutional review board of Jikei University School of Medicine approved the study.

## 3. Results

One hundred four patients aged 25 to 79 years (mean 58.8 years) were diagnosed with sinus fungus ball based on the histological findings. Female dominance was seen with 61 female patients (58.7%) and 43 male patients (41.3%). Major presenting symptom was purulent nasal discharge (35.6%) and facial pain (24.0%) (Table 1). CT was performed preoperatively for all cases, but complete data were available for only 100 cases. High density area in the affected sinus was seen in 82 cases (82%) (Table 2). Preoperative T2-weighted MR imaging was performed for 32 cases and showed low intensity area in the involved sinuses in all cases (100%) (Table 2).

The paranasal sinus localizations of fungus ball in the 104 patients are shown in Table 3. The most commonly involved sinus was the maxillary sinus (86/104, 82.7%) followed by the sphenoid sinus (11/104, 10.6%). Two patients had bilateral fungus ball in maxillary sinuses. Three patients with a history of transsphenoidal excision of pituitary macroadenoma had sphenoid fungus ball (3/11, 27.3%). Histological examination found that most of the fungus balls consisted of *Aspergillus* species (98/104, 94.2%) (Table 4). The sensitivity of culture study was low (11/66, 16.7%) (Table 5).

All cases were treated with endoscopic sinus surgery. The affected sinus was widely opened and the mass was meticulously removed. Edematous mucosa of the affected sinus was curetted leaving the basal membrane intact. The sinus was irrigated with normal saline according to the surgeon's preference.

Patients were instructed to perform nasal lavage with normal saline two times per day. The nasal cavity was examined with a rigid endoscope and secretions and crusts were cleaned at the outpatient department. Any small pieces of fungus ball in the operated sinus were removed immediately. Recurrence with occlusion of the operated sinus occurred in 3 (3.2%) of 94 patients who visited the outpatient clinic at least once.

## 4. Discussion

Sinus fungus ball is the most common form of fungal sinusitis. Fungal sinusitis is classified into two major categories, noninvasive fungal sinusitis and invasive fungal sinusitis [1, 8, 9]. Non-invasive fungal sinusitis is defined as absence of the fungal hyphae in the mucosa of the sinus and occurs in immunocompetent patients. This subtype is divided into fungus ball and AFS. Diagnosis of AFS is based on the detection of inspissated allergic mucin grossly at surgery. And histologically, the allergic mucin must be positive for fungal hyphae on fungal staining [10]. Invasive fungal sinusitis is defined as fungal sinusitis with mucosal infiltration of mycotic organisms and can be classified into three categories, granulomatous, acute fulminant, and chronic invasive, depending on the histological features [11, 12]. Invasive fungal sinusitis occurs in immunocompromised hosts or patients with diabetes mellitus, and the outcome is poor, especially for patients with the acute fulminant type.

Sinus fungus ball is mostly encountered in older individuals with the average age at presentation of 49 years (*n* = 173, France) [3], 52.7 years (*n* = 160, Italy) [2], and 61.1 years (*n* = 90, Taiwan) [6]. In our Japanese patients, the average age was 58.8 years (*n* = 104) with a range from 25 to 79 years. Female predominance has been consistent. The ratio of females is 60.1% (*n* = 173, France) [7], 66.1% (*n* = 109, France) [5], 73.8% (*n* = 160, Italy) [2], and 76.7% (*n* = 90, Taiwan) [6]. In this study, the ratio was 58.7%. The cause of female predominance remains unexplained but one possible reason is that fungus balls are more common in the older population and older women outnumber older men [13]. The life expectancy in the Japanese population was 79.6 years for males and 86.4 years for females in 2009 (http://www.mhlw.go.jp/toukei/saikin/hw/life/life09/sankou02.html). However, the numbers of male and female patients aged under 60 years in our series were the same (26 males and 26 females), so this idea is very plausible.

**Figure 1:**
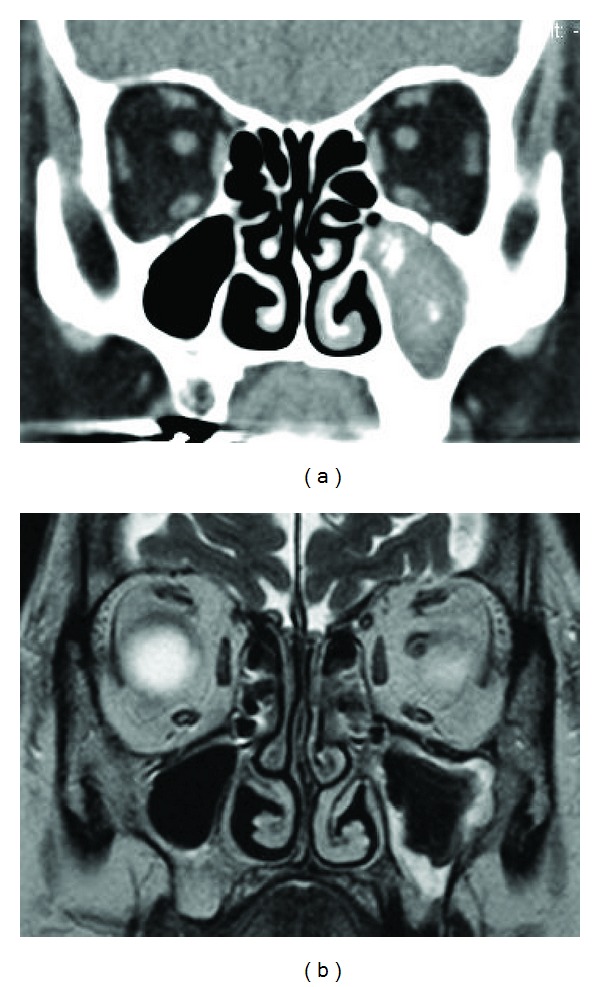
Typical neuroimaging findings of maxillary fungus ball. (a) Coronal CT scan with soft tissue density. Left maxillary sinus is completely filled with material. High density spots are seen. (b) Coronal T2-weighted MR image. Extremely low signal intensity to signal void indicates the presence of fungus ball. Peripheral high intensity area indicates edematous mucosa.

**Figure 2:**
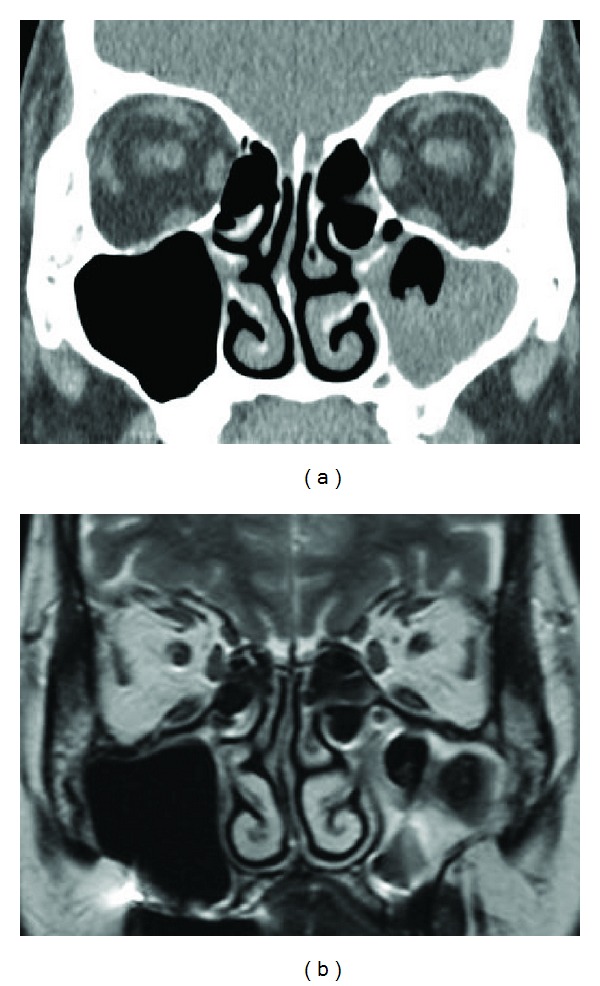
Representative case of fungus ball identifiable only on MR imaging. (a) Coronal CT scan with soft tissue density. Left maxillary sinus is filled with material. Irregular surface of the material suggests the possibility of fungus ball. High density spot is not seen. (b) Coronal T2-weighted MR image. Extremely low signal intensity to signal void indicates the presence of fungus ball.

Common CT findings include the following: ipsilateral involvement; bony thickening of the diseased sinus wall; and hyperdense area within the lesion (Figure 1). This high density is the consequence of the high content of heavy metals (iron and manganese) and calcium within the fungal hyphae and is extremely specific but lacks sensitivity [14, 15]. In our series, CT showed high density mass in 82% of cases. Heavy metals and calcium appear as a very low signal intensity to signal void on T2-weighted MR imaging (Figure 2). In our series, sensitivity was 100% (*n* = 32). In contrast to the specificity of high density on CT, low signal intensity on T2-weighted MR imaging is not specific to fungus ball. The signal patterns of eosinophilic mucin are similar to those of fungus ball. To distinguish fungus ball from eosinophilic chronic rhinosinusitis and AFS, the localization of the disease should be considered. Fungus ball is mostly isolated and unilateral, whereas eosinophilic chronic rhinosinusitis and AFS are diffuse and often associated with nasal polyp [14, 15]. Since the sensitivity of MR imaging was 100% in our series, we suggest that MR imaging is performed if CT demonstrates isolated and unilateral lesion without apparent calcification area.

In this study, the most common localizations were the maxillary sinus (82.7%) and the sphenoid sinus (10.6%) as in previous studies [2, 3, 5, 6]. The reason for this remains unexplained. Aerogenic theory suggests that the inhaled fungal spores are deposited in the sinuses, commonly the ethmoid sinus, and become pathogenic when the sinus begins to be anaerobic [2]. Fungal sinusitis may be regarded as a special form or complication of chronic recurring sinusitis [16]. However, ostiomeatal complex obstruction is not correlated with the growth of maxillary fungus ball which contradicts these hypotheses [17].

The causative fungus was mainly *Aspergillus* species, as shown by both histological examination and culture study as previously reported [2, 5, 7]. However, culture survey had extremely low sensitivity of 16.7%, as seen in previous studies ranging from 20.3% to 31.0% [2, 5, 7]. This difficulty in getting fungi to grow can be attributed to lack of viability of the fungus ball [13]. On the other hand, *Aspergillus* is a ubiquitous fungus found in nature. Culture of meticulously irrigated solutions of the noses of healthy people detected fungi in 100% of cases [18]. The value of culture study for the identification of fungus ball remains unclear.

The prognosis for sinus fungus ball is favorable. The reported recurrence rates are 0% (*n* = 160) [2], 0.6% (*n* = 173) [3], and 3.7% (*n* = 109) [5]. In our series, 3 of 94 patients (3.2%) had recurrence at 3, 5, and 6 months after operation. The recurrence was accompanied with occlusion of the operated sinus. Meticulous removal of the fungus ball, widening the drainage pathway, nasal irrigation at and after operation, and outpatient followup with endoscopic examination are necessary.

In conclusion, MR imaging provides high sensitivity but poor specificity for the identification of sinus fungus ball but is valuable for the investigation of undiagnosed cases detected with paranasal CT. The prognosis for fungus ball is very good, but recurrence is possible. Wide opening of the affected sinus and complete removal of the fungus ball are essential.

## Figures and Tables

**Table 1:** Chief complaint.

Symptoms	*N*	(%)
Purulent nasal discharge	37	(35.6)
Facial pain	25	(24.0)
Post nasal drip	14	(13.5)
Facial discomfort	9	(8.7)
Nasal obstruction	4	(3.8)

**Table 2:** CT and MR imaging findings.

	*N* (%)
High density area on CT	82/100 (82%)
Low intensity area on T2-weighted MR imaging	32/32 (100%)

**Table 3:** Paranasal sinus localizations.

	*N*	(%)
Maxillary sinus	86	(82.7)
Sphenoid sinus	11	(10.6)
Maxillary sinus and ethmoid sinus	5	(4.8)
Maxillary sinus and sphenoid sinus	1	(1.0)
Ethmoid sinus	1	(1.0)

Total	104	(100)

**Table 4:** Histological examination of fungus ball.

	*N*	(%)
*Aspergillus *	98	(94.2)
*Candida *	3	(2.9)
*Actinomycetes *	1	(1.0)
Unable to differentiate	2	(1.9)

Total	104	(100)

**Table 5:** Culture study of fungus ball.

	*N*	(%)
*Aspergillus* sp.	10	(15.2)
*Aspergillus* sp. + *Candida* sp.	1	(1.5)
Negative	55	(83.3)

Total	66	(100)
